# The effect of anti-PD-1/PD-L1 antibodies combined with VEGF receptor tyrosine kinase inhibitors versus bevacizumab in unresectable hepatocellular carcinoma

**DOI:** 10.3389/fimmu.2023.1073133

**Published:** 2023-01-23

**Authors:** Hui Zeng, Qi Xu, Jinyu Wang, Xiaoqing Xu, Jun Luo, Lei Zhang, Cong Luo, Jieer Ying, Jingjing Li

**Affiliations:** ^1^ Department of Interventional Radiology, Cancer Hospital of the University of Chinese Academy of Sciences (Zhejiang Cancer Hospital) Institute of Basic Medicine and Cancer (IBMC), Chinese Academy of Sciences, Hangzhou, Zhejiang, China; ^2^ Department of Hepato-Pancreato-Biliary & Gastric Medical Oncology, Cancer Hospital of the University of Chinese Academy of Sciences (Zhejiang Cancer Hospital) Institute of Basic Medicine and Cancer (IBMC), Chinese Academy of Sciences, Hangzhou, Zhejiang, China; ^3^ Medical Records and Statistics Department, Cancer Hospital of the University of Chinese Academy of Sciences (Zhejiang Cancer Hospital) Institute of Basic Medicine and Cancer (IBMC), Chinese Academy of Sciences, Hangzhou, Zhejiang, China; ^4^ The Second Clinical Medical College of Zhejiang Chinese Medical University, Hangzhou, Zhejiang, China; ^5^ Radiology Department, Cancer Hospital of the University of Chinese Academy of Sciences (Zhejiang Cancer Hospital) Institute of Basic Medicine and Cancer (IBMC), Chinese Academy of Sciences, Hangzhou, Zhejiang, China

**Keywords:** hepatocellular carcinoma, first-line treatment, immune checkpoint inhibition, VEGF receptor tyrosine kinase inhibitors, bevacizumab

## Abstract

**Introduction:**

Immune checkpoint inhibition (ICI) plus bevacizumab (BEV) is the standard first-line treatment for unresectable hepatocellular carcinoma (uHCC). We aimed to assess the efficacy and safety of ICI plus bevacizumab and ICI plus receptor tyrosine kinase inhibitor (TKI) in this patient population.

**Methods:**

This retrospective single-institution study enrolled 94 patients with uHCC who received ICI plus TKI or bevacizumab as the first-line treatment. Progression-free survival (PFS), overall survival (OS), objective response rate (ORR), and disease control rate (DCR) were used to evaluate treatment efficacy. RECIST v1.1 criteria were used to calculate the objective clinical response. Common Terminology Criteria for Adverse Events were used to report and categorize adverse events.

**Results:**

By the last follow-up interview on May 15, 2022, there were 57 deaths, and 19 patients did not develop disease progression. Thirty patients received sintilimab/atezolizumab plus bevacizumab (ICI + BEV group), and 64 received ICI plus TKI (ICI + TKI group). The median OS was 430 days (95% CI, 266-NA) in the ICI+TKI group and 498 days (95% CI, 349-NA) in the ICI+BEV group (HR, 1.20; 95% CI, 0.69-2.07; P = 0.52). There was no significant difference between the two groups in the median PFS (182 vs. 221 days, P=0.67). In the ICI+TKI group, the ORR and DCR were 28.1% and 67.2%, respectively. In the ICI+BEV group, the ORR and DCR were 26.7% and 66.7%, respectively. The overall incidence of adverse events was similar between the two groups. Palmar-plantar erythrodysesthesia syndrome (23[36%]) occurred only in the ICI + TKI group. Patients who received ICI+BEV were more prone to upper gastrointestinal bleeding (2 [7%]), with one patient with grade 4 requiring emergency DSA treatment.

**Conclusion:**

This study found that ICI+TKI and ICI+BEV as first-line treatments were similar in OS, PFS, and tumor response in uHCC. Different populations are suitable for different regimens because of the different adverse events.

## 1 Introduction

Hepatocellular carcinoma (HCC) is the most common malignancy worldwide, ranking 6^th^ in incidence and 3^rd^ in mortality in 2020 (1, 2). In China, the incidence and mortality rates of HCC are higher than the global average (1). The success of the SHARP study led to an era of targeted therapy for unresectable HCC (uHCC), and sorafenib has become the standard first-line treatment for uHCC (3). However, due to limited efficacy and intolerable treatment-related adverse events, the overall survival of patients with uHCC is poor and has not improved over the last decade. The KEYNOTE-224 (4) and CheckMate-040 (5) studies indicated that immune checkpoint inhibition (ICI) might be a treatment option for patients with uHCC. After the failure of the KEYNOTE-240 (6) and CheckMate-459 studies (7), a new therapeutic mode of immunotherapy in uHCC needs to be explored. Some studies have demonstrated that a combination of ICI and vascular endothelial growth factor (VEGF) receptor tyrosine kinase inhibitors (TKI) could prolong the survival of patients with uHCC (8, 9). Meanwhile, the IMbrave 150 (10) and Oriental-32 studies (11) showed that ICI plus bevacizumab, a monoclonal antibody that binds VEGF, improved the prognosis of patients with uHCC compared with that using sorafenib alone. However, no randomized controlled trials or retrospective studies have been conducted to compare the efficacy of ICI plus bevacizumab or ICI plus TKI in patients with uHCC. In this study, we aimed to compare the efficacy and safety of ICI plus VEGF receptor (VGFR) TKI or bevacizumab as first-line treatment for uHCC and explore the potential advantages of the two models.

## 2 Materials and methods

### 2.1 Study population

Patients with uHCC who received ICI plus VEGFR TKI or bevacizumab therapy at Zhejiang Cancer Hospital from January 2018 to June 2021 were enrolled in this study. Eligible patients were pathologically or clinically diagnosed with u HCC (not amenable to curative surgery or local treatment). The patients had not previously received systemic therapy for uHCC before receiving ICI plus VEGFR TKI or bevacizumab therapy. Patients received at least one dose of the study drug and underwent at least one post-baseline radiographic tumor response assessment according to the Response Evaluation Criteria in Solid Tumors, version 1.1 (RECIST 1.1). Patients lost to follow-up and those who had no clinical efficacy evaluation were excluded. All patients were followed up to the date of death or May 15, 2022. Clinical information, disease stage, drug information, and adverse events (AEs) were collected and recorded. The study complied with all relevant ethical regulations and was approved by the Institutional Review Board (IRB) and Ethics Committee (EC) of Zhejiang Cancer Hospital (approval no. IRB-2022-332(ke)).

### 2.2 Treatment

Treatment information, including the treatment start date, drug name, drug dose, laboratory data, radiological evaluation, and treatment-emergent AEs, was systematically recorded. The dosage of ICIs was administered intravenously according to the instructions; pembrolizumab, sintilimab, camrelizumab, and tislelizumab were administered at a dosage of 200 mg every 3 weeks, and atezolizumab was administered at a dosage of 1200 mg every 3 weeks. Bevacizumab was administered at a dose of 15 mg/kg every 3 weeks. No dose reduction was permitted for ICI or bevacizumab. The VEGFR-TKI drugs included lenvatinib, sorafenib, and apatinib. Lenvatinib, sorafenib, and apatinib were administered orally once daily at an initial dosage of 8 mg, 400 mg, and 250 mg, respectively.

### 2.3 Outcome assessment

Patients were followed up on May 15, 2022. Patients who did not experience disease recurrence at the time of the last follow-up or who had died were censored at the last follow-up or date of death, respectively. RECIST v1.1 criteria were used to calculate the objective clinical response the professional radiologists evaluated at the center of Zhejiang Cancer Hospital. Computed tomography/magnetic resonance imaging (CT/MRI) was performed every six weeks to assess treatment response. Progression-free survival (PFS), overall survival (OS), objective response rate (ORR), and disease control rate (DCR) were used to evaluate treatment efficacy. Safety data were continuously assessed by monitoring adverse events, laboratory testing, vital signs, and physical examination. The Common Terminology Criteria for Adverse Events (CTCAE, version 4.0) were used to report and categorize adverse events.

### 2.4 Statistical analysis

Numerical data are described as mean ± SD and differences between groups were compared using the t-test. Categorical data are described by ratios, and the chi-square test was conducted to compare the differences between groups. The Kaplan–Meier method was used to estimate OS and PFS, and the log-rank test was used to analyze the comparisons. Cox proportional hazard modeling was used to estimate the hazard ratios (HRs) of each clinicopathological feature for PFS and OS. Univariate and multivariate logistic regression models were used to determine the predictors associated with ICI + TKI or ICI + BEV treatment response. Statistical significance was set at P < 0.05. Statistical analyses were performed using R-3.6.3 software.

## 3 Results

### 3.1 Baseline characteristics of all patients

**Figure 1:**
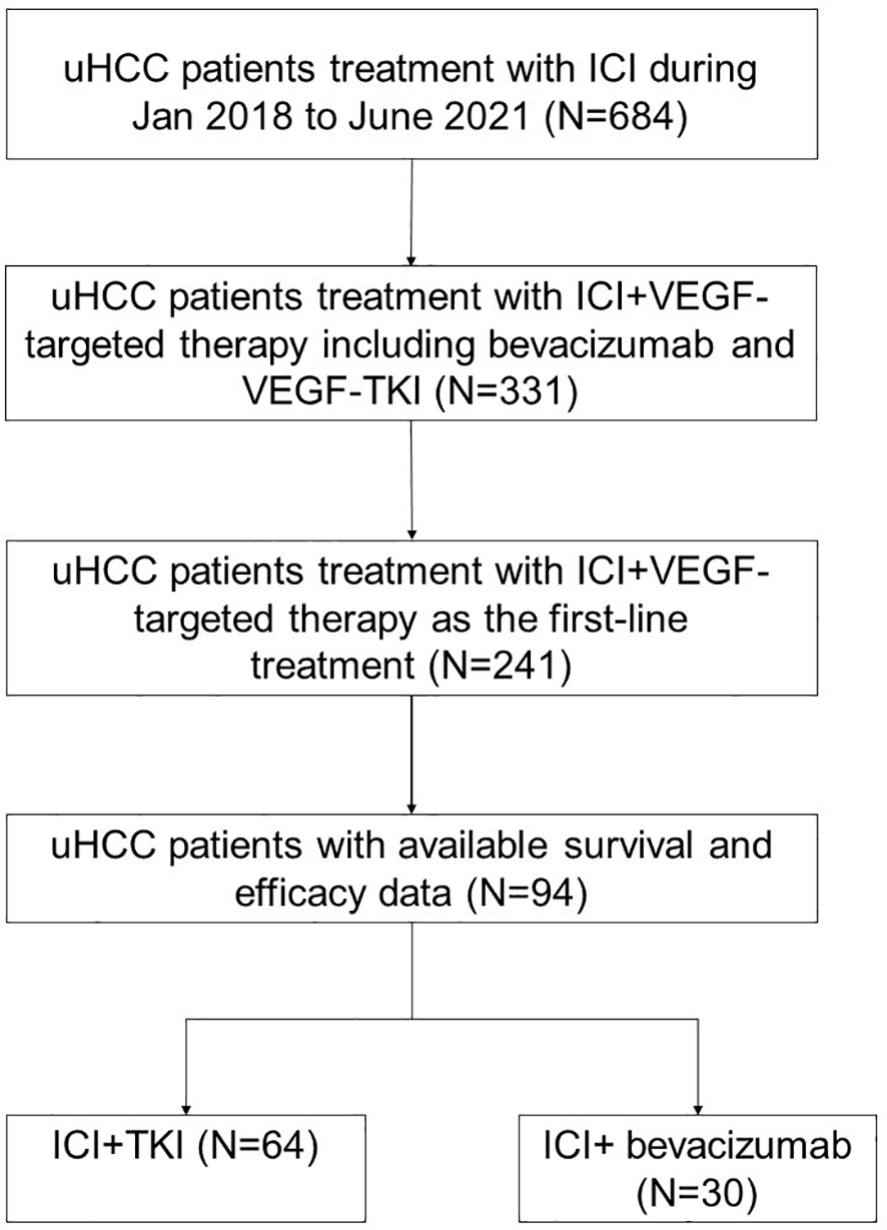
Trial profile.

A total of 94 patients with uHCC who received ICI plus VEGFR TKI or bevacizumab as first-line treatment were included in this study (**Figure 1**). Thirty patients received sintilimab/atezolizumab plus bevacizumab treatment (ICI + BEV group). In comparison, 64 patients received anti-PD-1 antibodies plus VEGFR TKI therapy (ICI + TKI group). In the ICI+TKI group, anti-PD-1 antibodies included pembrolizumab, sintilimab, camrelizumab, and tislelizumab; TKIs included sorafenib, lenvatinib, and apatinib. The demographic and baseline characteristics of the 94 patients are summarized in **Table 1**. In the ICI+TKI group, the median age was 57.6 years, and 87.5% were male; in the ICI+BEV group, the median age was 53.7 years, and 86.7% were male. Most patients were infected with HBV (87.5% in the ICI+TKI group and 93.3% in the ICI+BEV group). The ECOG performance score (ECOG PS), Barcelona Clinic Liver Cancer (BCLC) stage, portal vein tumor thrombus (PVTT), extrahepatic metastases (EHS), sites of metastasis, and ascites were well balanced between the two groups. The high AFP level group (AFP ≥ 200 ng/mL) and low AFP level group (AFP < 200 ng/mL) were also similar between the two groups. More patients in the ICI + BEV group had prior surgery.

**Table 1:** Baseline Characteristics of the Study Participants.

Characteristic	ICI+TKI Group (N=64)	ICI+BEV Group (N=30)	P
**Male sex, n (%)**	56 (87.5)	26 (86.7)	1
**Median age (range), years**	57.6 (25-83)	53.7 (32-73)	0.107
**Viral etiology, n (%)**			0.641
** HBV**	56 (87.5)	28 (93.3)	
** HCV**	3 (4.7)	1 (3.3)	
** Nonviral**	5 (7.8)	1 (3.3)	
**ECOG PS, n (%)**			0.579
** 0**	19 (29.7)	12 (40.0)	
** 1**	39 (60.9)	15 (50.0)	
** 2**	6 (9.4)	3 (10.0)	
**BCLC, n (%)**			1
** B**	8 (12.5)	4 (13.3)	
** C**	56 (87.5)	26 (86.7)	
**PVTT, n (%)**	24 (37.5)	8 (26.7)	0.356
**EHS, n (%)**	35 (54.7)	20 (66.7)	0.37
**AFP≥200 ng/ml, n (%)**	28 (43.8)	18 (60.0)	0.185
**Prior therapy, n (%)**			
** surgery**	18 (28.2)	17 (56.7)	0.011
** TACE**	35 (54.7)	19 (63.3)	0.505
** radiotherapy**	8 (12.5)	0	0.052
**Sites of metastasis**			
** Liver**	32 (50.0)	12 (40.0)	0.385
** Lung**	19 (29.7)	15 (50.0)	0.068
** Lymph nodes**	15 (23.4)	5 (16.7)	0.592
** Peritoneum**	7 (10.9)	3 (10.0)	1
** Others**	8 (12.5)	0	0.052
**Ascitic**	13 (20.3)	4 (13.3)	0.568
**Sintilimab**	35	21	–
**Atezolizumab**	0	9	–
**Pembolizumab**	13	0	–
**Camrelizumab**	6	0	–
**Tislelizumab**	10	0	–
**Sorafenib**	17	0	–
**Lenvatinib**	41	0	–
**Apatinib**	6	0	–
**Bevacizumab**	0	30	–

HBV, Hepatitis B virus; HCV, Hepatitis C virus; ECOG PS, Eastern Cooperative Oncology Group performance status; BCLC= Barcelona Clinic Liver Cancer stage; PVTT, portal vein tumor thrombus; EHS, extrahepatic metastases.

### 3.2 Treatment and efficacy

**Figure 2:**
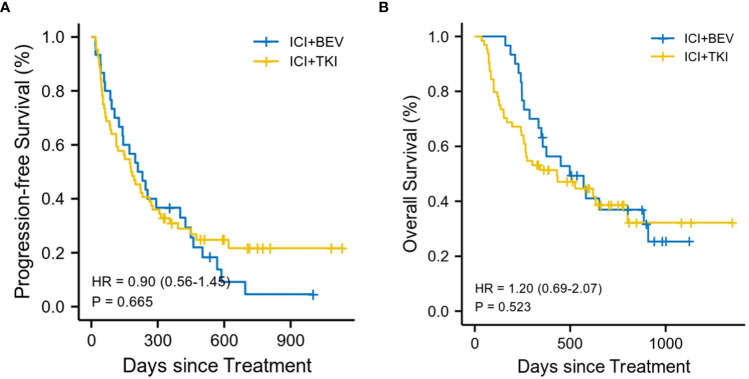
Kaplan-Meier estimates of progression-free and overall survival **(A)** Progression-free survival. **(B)** Overall survival.

By the last follow-up interview on May 15, 2022, of the total of 94 patients, there were 57 deaths, including 37 patients in the ICI+TKI group and 20 patients in the ICI+BEV group, with a median follow-up for overall survival of 450 days. Nineteen patients (20.2%) did not show disease progression, including 16 and 3 patients in the ICI+TKI and ICI+BEV groups, respectively. In the ICI+TKI group, 34 patients died of disease progression and 3 of unknown causes. In the ICI+BEV group, 18 patients died of disease progression and 2 of unknown causes. No treatment-related deaths occurred in this study. The median OS was 430 days (95% CI, 266 to NA days) in the ICI + TKI group and 498 days (95% CI, 349 to NA days) in the ICI + BEV group (HR, 1.20; 95% CI, 0.69 to 2.07; *P* = 0.52; shown in **Figure 2A**). The median PFS was 182 days (95% CI, 113–227 days) in the ICI+TKI group and 221 days (95% CI, 141–449 days) in the ICI+BEV group (HR, 0.90; 95% CI, 0.56 to 1.45; *P* = 0.67; shown in **Figure 2B**). Eighteen (28.1%) and 8 (26.7%) patients in the ICI+TKI and ICI+BEV groups, respectively, achieved an objective response. Of these patients, one CR was observed in each group, and the remaining patients showed PR. In total, there were 25 (39.1%) and 12 (40%) patients in the ICI+TKI and CI+BEV groups, respectively. Therefore, the overall radiologically confirmed ORR and DCR were 28.1% and 67.2%, respectively, in the ICI + TKI group, and 26.7% and 66.7%, respectively, in the ICI + BEV group (**Table 2**).

**Table 2:** Tumour responses according to RECIST version 1.1.

Response parameters	ICI+TKI Group	ICI+BEV Group	P
	(N=64)	(N=30)	
**Complete response, n**	1	1	
**Partial response, n**	17	7	
**Stable disease, n**	25	12	
**Progressive disease, n**	21	10	
**Objective response rate, n (%)**	28.1	26.7	0.545
**Disease control rate, n (%)**	67.2	66.7	0.570

### 3.3 Subgroup analyses

**Figure 3:**
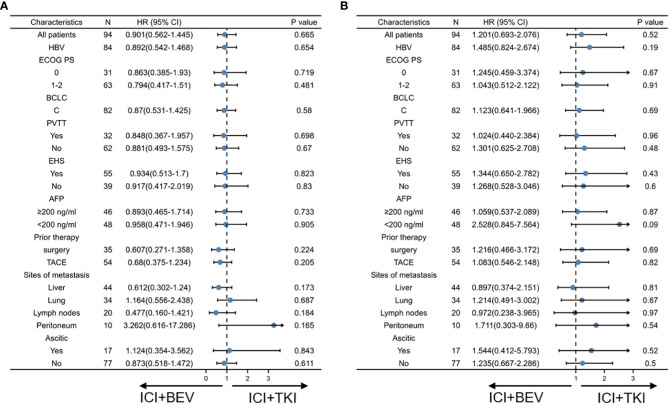
Subgroup analysis of progression-free survival and overall survival in intention-to-treat population at final analysis. **(A)** Progression-free survival. **(B)** Overall survival.

To determine whether a specific patient population could benefit from ICI+TKI or ICI+BEV, we conducted a subgroup analysis (shown in **Figure 3**). An exploratory multivariate analysis using a Cox proportional hazards model identified no statistically significant differences in OS and PFS between subgroups.

### 3.4 Safety

All treatment-related adverse events with an incidence of > 5%, or grades 3-4, are shown in **Table 3**. In total, all patients who received ICI+TKI and 29 (97%) of 30 patients who received ICI+BEV experienced an adverse event. The overall incidence of adverse events was similar between the two groups; however, adverse events differed between the ICI+TKI and ICI+BEV groups. Fatigue was the most common adverse event in both groups (51 [80%] vs. 25 [83%]), with most being grade 1-2 and with no significant difference between the two groups. Palmar-plantar erythrodysesthesia syndrome occurred only in the ICI + TKI group, with an incidence rate of 36%; there was no palmar-plantar erythrodysesthesia syndrome in the ICI + BEV group. Of note, patients who received ICI + BEV were more prone to upper gastrointestinal bleeding (2 [7%]), and one patient with grade 4 bleeding required emergency DSA treatment.

**Table 3:** Common treatment-related adverse events.

	ICI+TKI Group (N=64)		ICI+BEV Group (N=30)	
AE	Any grade	Grade 3-4	Any grade	Grade 3-4
**Any**	64 (100%)	29 (45%)	29 (97%)	11 (37%)
**Fatigue**	51 (80%)	4 (6%)	25 (83%)	2 (7%)
**Palmar-plantar erythrodysaesthesia syndrome**	23 (36%)	8 (12%)	0	0
**Increased ALT or AST**	34 (53%)	1 (2%)	16 (53)	0
**Proteinuria**	28 (44%)	5 (8%)	14 (47%)	2 (7%)
**Decreased platelet count**	22 (34%)	5 (8%)	9 (30%)	4 (13%)
**Abdominal pain**	14 (22%)	0	6 (20%)	0
**Hypothyroidism**	11 (17%)	0	7 (23%)	0
**Pruritus**	10 (16)	0	5 (17%)	0
**Increased blood bilirubin**	9 (14%)	3 (5%)	4 (13%)	1 (3%)
**Hypertension**	7 (11%)	4 (6%)	5 (17%)	3 (10%)
**Rash**	7 (11%)	0	2 (7%)	0
**Diarrhea**	6 (9%)	0	3 (10%)	0
**Nausea**	5 (8%)	0	2 (7%)	0
**Decreased neutrophil count**	5 (8%)	1 (2%)	3 (10%)	0
**Vomiting**	4 (6%)	0	2 (7%)	0
**Pneumonia**	1 (2%)	1 (2%)	0	0
**Upper gastrointestinal haemorrhage**	0	0	2 (7%)	1 (3%)

## 4 Discussion

To our knowledge, data on head-to-head comparisons across ICIs combined with VEGFR TKI and bevacizumab as first-line treatment for uHCC are sparse. This study is the first to compare the efficacy and safety of ICI plus VEGFR TKI and ICI plus bevacizumab as first-line treatment in patients with uHCC.

In 2008, the SHARP trial (3) demonstrated the superiority of sorafenib over placebo (OS, 10.7 months versus 7.9 months), which was a breakthrough in uHCC systemic therapies. Sorafenib has been the primary standard for uHCC for a decade. ICIs have shown significant therapeutic effects in various cancers. In the phase 2 KEYNOTE-224 trial (4), pembrolizumab was effective and tolerable in patients with uHCC who had previously been treated with sorafenib. Another phase 1/2 CheckMate 040 (5) trial showed that nivolumab could increase tumor reduction and ORRs of 15–20% in patients with uHCC. Compared with previous data on sorafenib, ICI showed better efficacy and safety in uHCC and is expected to become a new treatment regimen. However, the phase 3 KEYNOTE 240 trial (6) failed to show a statistically significant superiority of pembrolizumab over placebo in terms of OS and PFS. Concurrently, in another phase 3 trial Checkmate 459 trial (7), nivolumab treatment also did not significantly improve OS compared with sorafenib (15.2 months versus 13.4 months). Both the KEYNOTE 240 and CheckMate 459 trials failed, possibly related to the study endpoint set as OS. Under the existing conditions, the proportion of patients with uHCC receiving second-line and later- line treatment has increased significantly (12), which has had a great impact on OS. Nevertheless, the clinical activity and favorable safety profile of ICIs have been observed in patients with uHCC.

Subsequently, the IMbrave 150 study showed that atezolizumab, an anti-PD-L1 antibody, plus bevacizumab had better outcomes in patients with uHCC than sorafenib, setting a new first-line median OS duration of 19 months (10). Similarly, the anti-PD-1 antibody sintilimab plus bevacizumab versus sorafenib improved OS in Chinese patients (11). The treatment mode of ICI plus TKI is widely used in clinical practice. Several previous small-sample studies have shown that ICI plus TKI improves the survival of patients with uHCC, including pembrolizumab plus lenvatinib, camrelizumab plus apatinib, and nivolumab plus lenvatinib (8, 9, 13). By 2022, the European Society for Medical Oncology Congress phase 3 LEAP-002 trial showed that, compared with lenvatinib, pembrolizumab plus lenvatinib did not meet its dual primary endpoints of OS and PFS as a first-line treatment for patients with uHCC (14). However, there were trends toward improvement in OS and PFS for patients who received pembrolizumab plus lenvatinib versus those who received lenvatinib alone. Sorafenib was used as controls for IMbrave150 and Orient-32, while that for the LEAP-002 was lenvatinib. Lenvatinib was marketed globally in the REFLECT study, a non-inferiority designed phase III clinical trial, and has become the standard first-line treatment for advanced liver cancer with sorafenib (15). In the REFLECT study, lenvatinib had a slight advantage over sorafenib in OS (HR=0.92, 95%CI: 0.79-1.06) but had a considerable advantage in PFS (HR=0.66, 95% CI: 0.57-0.77). In addition, a network meta-analysis showed that the estimated median HR for OS and PFS of atezolizumab plus bevacizumab versus lenvatinib was 0.63 (95% CI 0.39-1.04) and 0.91 (95% CI 0.42-1.99) (16), which was similar to those observed by Sonbol (17). In other words, ICI+TKI is not superior to lenvatinib but may be superior to sorafenib. This was proven in a phase 3 study at the 2022 European Society for Medical Oncology Congress. The trial showed that camrelizumab (anti-PD-1 IgG4 monoclonal antibody) plus rivoceranib (apatinib; VEGFR2-TKI) significantly prolonged PFS and OS and improved ORR in uHCC compared to sorafenib (18).

The choice of ICI+BEV or ICI+TKI for the first-line treatment of patients with uHCC is a practical problem that is a challenge for clinicians. Our study collected two relatively matched groups of patients at baseline from one center during the same period and compared the efficacy of ICI plus TKI and ICI plus bevacizumab in the first-line treatment of uHCC. The results showed no statistical differences between the two groups in terms of OS, PFS, and ORR. In the subgroup analysis, the results of the Kaplan–Meier analysis showed no statistically significant difference between the two groups. This suggests that the short-term and long-term efficacies of ICI+TKI and ICI+BEV in the first-line treatment of uHCC are similar.

The main difference between the two groups was the combination of drugs with ICI namely, bevacizumab in one group and receptor TKI in the other. Bevacizumab is a recombinant human monoclonal IgG1 antibody that acts by inhibiting the biological activity of human VEGF (19). The TKI drugs used in this study included sorafenib, lenvatinib, and apatinib. Although the target sites of these drugs include FGFR, PDGFR, and KIT, the main target site of these drugs is still VEGFR (20–22). In tumor tissues, VEGF released by hypoxic cancer cells and vascular endothelial cells plays an important role in promoting tumor growth, invasion, and metastasis. VEGF can enhance the activity of regulatory T cells (Tregs), promote the release of immunosuppressive cytokines, and mobilize tumor-associated macrophages (TAMs) to promote their polarization toward the M2 phenotype (23–25). VEGF also activates myeloid-derived suppressor cells (MDSCs), which in turn release more VEGF (26). VEGF-induced Tregs, TAMs, and MDSCs reduce the proliferation and function of CD8+ cells. VEGF also prevents antigen-activated CD8+ cells from infiltrating tumor tissues through its effect on tumor angiogenesis (27). Bevacizumab and molecular- targeted drugs can inhibit VEGF activity. These agents increase antigen presentation by dendritic cells, promote T-cell activation in the priming phase, and improve T-cell migration from lymph nodes to tumor sites by normalizing the tumor blood vessels (28, 29). In addition, these agents have been found to inhibit the production of Tregs, TAMs, and MDSCs at tumor sites and to negatively regulate the expression of immunosuppressive cytokines such as TGF-β and IL-10 (30, 31). Therefore, administration of PD-1/PD-L1 antibody under these conditions could enhance the antitumor activity of T cells, which explains the synergistic effect of ICI combined with VEGFR-targeting drugs. Molecular targeted drugs, such as lenvatinib, can inhibit FGFR1–FGFR4, RET, KIT, and PDGFRα, which can also increase the efficacy of ICI (32, 33), but their effects may be limited and cannot play a decisive role in the treatment of uHCC.

In addition to the efficacy, the treatment-related adverse events also deserve special attention. In our study, most patients in both groups experienced adverse events. Common treatment-related adverse events in both groups included fatigue, increased ALT or AST, proteinuria, and decreased platelet count, consistent with previous studies (10, 11, 34). There were no significant differences in immune-related adverse events between the two groups. However, the incidence of grade 3/4 serious adverse events in the ICI + TKI group was higher than that in the ICI + BEV group. The main difference was that the incidence of palmar-plantar erythrodysesthesia syndrome in the ICI+TKI group was 12%, which has been observed in previous studies (3, 15, 35). In contrast, no such adverse events were observed in the ICI + BEV group. Severe palmar-plantar erythrodysesthesia syndrome has a significant impact on the patients’ quality of life. Therefore, patients in the IC+BEV group had better self-perception. However, it is of particular concern that patients who received ICI + BEV were at a greater risk of upper gastrointestinal hemorrhage. In this study, two patients in the ICI+BEV group developed complications related to upper gastrointestinal hemorrhage. Among them, a patient with cirrhosis and severe esophagogastric varices detected by esophagogastroduodenoscopy developed life-threatening upper gastrointestinal bleeding after ICI+BEV treatment and received emergency DSA treatment. The patient did not restart IC + BEV treatment. A real-world study found no correlation between the grade of varices at pretreatment esophagogastroduodenoscopy and bleeding events (36). The main effect of bevacizumab is inhibition of the VEGF signaling pathway. VEGF plays a vital role in maintaining the architecture and integrity of the microvasculature of endothelial cells. When bevacizumab blocks VEGF signaling, endothelial cells are impaired in their ability to repair and renew wounds, leading to an increased risk of bleeding (37, 38). In addition, some studies have found that the risk of bleeding is positively correlated with the dose level of bevacizumab (39), and the dose of bevacizumab in HCC is relatively high (15 mg/kg); therefore, the risk of bleeding cannot be ignored. We still need to carefully choose bevacizumab in such patients to prevent life-threatening upper gastrointestinal bleeding.

Our study has some limitations. First, it was retrospective in nature, with the possibility of selection bias. Atezolizumab plus bevacizumab, or sintilimab plus bevacizumab, is expensive, and the patients in this group were better off financially than those in the ICI + TKI group. Bevacizumab was previously associated with an increased risk of bleeding in patients with uHCC with significant cirrhosis; therefore, these patients avoided bevacizumab. Second, patients were only selected from one hospital, and the results may be influenced by practices specific to that hospital. Third, this study had a small sample size and few events. Fourth, the drugs used in this study were diverse, especially in the ICI+TKI group, which included four ICIs and three TKIs. These limitations may have had an impact on the results of this study. More studies, especially phase III randomized controlled trials, are needed to further validate the results of this study.

## 5 Conclusion

We compared the efficacy and safety of ICIs combined with VEGFR TKI and ICIs combined with bevacizumab as first-line treatments for uHCC. Our research found that the two treatment regimens were similar in OS, PFS, and tumor response. Concurrently, there were some differences in the adverse events between the two treatment regimens. Therefore, this observation may provide a significant reference for future clinical practice.

## Data availability statement

The raw data supporting the conclusions of this article will be made available by the authors, without undue reservation.

## Ethics statement

The studies involving human participants were reviewed and approved by Institutional Review Board (IRB) and Ethics Committee (EC) of Zhejiang Cancer Hospital. The ethics committee waived the requirement of written informed consent for participation.

## Author contributions

Concept and design: JJL and HZ. Recruitment and management of patients and approval of final manuscript: JJL, QX, JW, XX, JL, LZ, CL, JY, and HZ. Statistical analysis: JW and JJL. Drafting of manuscript: JJL, XX, JW, and HZ. All authors contributed to the article and approved the submitted version.
